# Remarks, Gleanings, and Extracts

**Published:** 1874-12

**Authors:** 


### Remarks, Gleanings and Extracts.

---

BY ALBAN S. PAYNE, M.D., OF VIRGINIA.

*Excoriated Nipples.*—In primipara this is often the exciting cause of serious after-trouble with the breast, and usually arises from a poorly developed nipple. The nipple is so small and one sided, and the child, small and weak, that it is impossible for the child to nurse its mother. The child in its repeated inffectual efforts to catch and hold the imperfectly developed nipple, produces irritation, the milk accumulates; irritation becomes inflammation; pus quickly follows, and, after intense suffering to the mother, the breast has to be lanced, it may be more than once this operation has to be repeated. Now, I think much trouble and suffering might be prevented if every young and newly married woman would, at least six months prior to her confinement, bath her breast with cold water, wipe dry, and pull her nipples, "yes milk them" regularly night and morning. This plan carried out faithfully would both toughen and develop the "nipples," so that at the birth of her child, it would find no difficulty of nursing its own mother. For sore nipples there are several valuable recipes. Tannin, grs. xv.; glycerin, qs., to thoroughly dissolve the tannin is a favorite prescription with some practitioners. With diseased nipples you will often find the child has the "thrush". For this reason, I prefer the following, as it is alike beneficial to the mother of the child, and to the nipple of the mother. ℞.—Cretæ preperatæ, grs. xx.; biborate soda, grs. xxx.; spts. rosemary, ℥ii.; alcohol, aqua pura, aa ℥ii. Mixed, and wash nipple immediately after each nursing of the child. In patients with sore nipples and suppurating mammary glands you will often find a scrofulous diathesis. In such cases the iodic remedies combined with ext. sarsaparilla, or given in an infusion of the common burdock, are required to effect a cure.

*Guavana for the Cure of Sick Headache.*.—I did not use the extract recommended by Professor Trousseau, but gave the powder in doses of ten to fifteen grains, repeated every two hours, and the patients rarely took three doses before they were relieved. A ten-grain dose taken immediately before the attack always prevented it. Some of my patients, who suffered every month from sick-headache, were entirely rid of their sufferings for several months, and some for one and two years after using one or two boxes of the powder, each box containing a dozen packages of ten grains.—*C. P. Carkout, Lockport, La., Baltimore Physician and Surgeon.*

Should the above prove literally true, it will be of inestimable value to the human family, whole families being subject to regular attacks of sick-headache.

*Picric Acid as a Test for Albumen.*—M. Galippe recommends, as an extremely sensitive and thoroughly reliable test for albumen, a saturated solution of picric acid. In nomal urine this solution never causes precipitation, while the most minute traces of albumen are readily detected by it. The *modus operandi* of applying the test is as follows: A drachm or two of the picric acid solution is put in a test tube, and a few drops of the fluid to be tested are allowed to fall into the solution. If albumen is present, it traces a characteristic white line through the testing solution.

---

### REMARKS, GLEANINGS AND EXTRACTS

BY THE LOCAL EDITORS.

*Villate's Mixture and Its Uses.*—The introduction of Villate's mixture in the surgical therapeutics is of recent origin. A French veterinary surgeon having stated that, with the use of this solution, he daily cured caries of bones in animals, and especially in the horse, Dr. Notta first thought of applying the remedy to the human subject, and in March, 1863, he published six observations.—The celebrated Nélaton heard of the result, and gave it a trial in his extensive practice, both in the hospital and outside. His successes were such as to bring this new therapeutical agent to the notice of the medical world.

In March, 1866, Dr. Notta published two memoirs confirmatory of these assertions, which proved to be worthy of a premium from the Academy of Medicine and a reward of three thousand francs.

It was in January, 1829, that Villate, the author of this solution, made known his first successes.

In 1831, Mr. Miroud gives the formula of the mixture of Mr. Villate, and says: "I have had several times the opportunity of observing its salutary effects in cases of caries. I noticed that it hastened the exfoliation of the necrosed or carious parts, gave a more healthy appearance to discolored surfaces, and had a tendency to stop certain morbid exhalations.

During the ensuing ten years no mention is made of this preparation. Some practitioners used it, but never published the results of their observations.

Up to 1842 the operation on the horse for fistulous withers was very frequently performed; but from that time, and since the publication of some very good observations on the use of this mixture injected in the fistulas resulting from caries of the fibro-cartilage of the bone of the foot (javart cartilagineux), that operation was altogether put aside.

From this date the solution became generally known, and the reputation of a few eminent veterinary surgeons is due solely to the rapid cures obtained by the use of this preparation.

They employed it against denuded surfaces, fistulas, caries, necrosis, profuse secretions, catarrhs of the ear, and some skin diseases of long standing. They always observed that the greater the chronciity the more satisfactory was the result. Its use was to be kept up until complete recovery. Even in cases where instruments had to be used for the removal of a large sequestrum, the topical application of this agent subsequent, as also previous to the operation, has always proved itself superior to all other known substances.

It has been used in caries of almost every bone and articulation of the body; in cold abscesses of the neck, deltoid region, back, superior third of the thigh; in fistules resulting from abscesses by congestion; those of the lachrymal gland, of the anus, of tuberculous affections of the testicle, etc. Diluted in water, one part to ten, it is said to cure every case of gleet.

Though I am inclined to believe that the efficacy of the mixture has been exaggerated by its advocates, still I do not doubt of the accuracy of the observations gathered and published, and think it a good addition to our therapeutics. Lately, in Paris, Dr. Polaillon cured several cases of chronic otorrhœa with this solution. The facts were so evident, the treatment so simple, that I concluded to use it in such cases, should I have an opportunity. During the last two years I have used it successfully four times; and in my researches, having failed to find any mention made by American physicians where this preparation has been used, I concluded to present to the Association my observations, with a few general remarks on the mixture.

The original formula of this solution, as first composed by Villate, is as follows:

℞. Liquoris plumbi subacetatis, ℥ i.; zinci sulphatis, crystal, and cupri sulphatis, crystal, aa ℥ ss.; aceti vini albi, fl. ℥ vjss. Mix. Dissolve the salts in the vinegar and add the subacetate of lead. Shake before using.

Dorvault, Bouchardat, and some other authors, put ℥viij. of vinegar instead of ℥ vjss ; but Dr. Notta does not think that this modification is of any advantage, and prefers Villate's original formula.

It is very important that this preparation should be made as I stated. Druggists very often substitute for the white-wine vinegar a solution of pyroligneous acid, in which case the liquid acts like a powerful caustic, and the patient cannot bear its application.— These two solutions can be very easily distinguished at first sight: when the pyroligneous acid is used, the solution, once settled, has a bluish hue; but when prepared with the white-wine vinegar it has a greenish hue. This is a capital point, for surgeons have noticed a great difference in using both preparations on the same patient. The pyroligneous acid solution has produced excessive pains and serious symptoms of irritation and inflammation.

I do not understand the idea of Villate in combining such substances, for the result is a general decomposition.

Evidently the mixture of Villate owes its precious qualities in therapeutics to the presence of all those substances entering into its composition, and not to any special one to the exclusion of the others. Each of these salts tried alone acts more or less like an astringent or a caustic, but does not give the same results; therefore, however strange seems to be the preparation, it is preferable not to modify it, as some have propsed to do.

The mixture of Villate, when first injected into a fistule, or applied to a wound, produces a sharp pain which may last an hour or more; but the patient soon becomes accustomed to it, and in a few days bears it without complaining. To avoid violent pains in nervous or irritable patients, it should be at first diluted with water, and the dilution gradually made stronger until they can bear it pure. The first injections determine inflammation in the parts coming in confact with the solution. Those inflammatory symptoms are generally limited. Suppuration is more abundant, but will soon diminish and stop entirely, which indicates a rapid process of cicatrization. In caries, flakes of bones will very often be washed out by the injections, or thrown out with the suppuration, but after their elimination the cure will soon follow.

Judging from the effects produced, the mixture of Villate seems to act as a mild caustic in stimulating the wounds, and sometimes in forming on the surface a thin eschar, or a false membrane, which, when removed, leaves a healthy and granulating tissue ready for cicatrization. This escharotic action in some cases may be too active, therefore it is necessary to watch the effects of the mixture, and not to allow it to remain in the bottom of wounds. The mixture of Villate could not be used, like tincture of iodine, in the treatment of cysts or circumscribed collections; in other words, in cavities not communicating with the exterior, into which more or less tincture of iodine can be safely injected. It is necessary that it should run out easily; it should, therefore, be employed only in the treatment of those cavities communicating with the exterior by means of fistulas.

The effects of these injections are local. Some authors, however, declare that when the injections stop profuse suppurations, the modification brought over the local affections is such that patients recuperate very fast, appetite and strength are restored, and they themselves call the attention of the surgeon to the change.—Dr. R. R. HOPKINS, *in the New-Orleans Med. & Surg. Journal.*

*Emulsio Carnis.*—Dr. James Kemble (*American Journal of Pharmacy*) concludes a valuable article on "raw beef" as follows:

It will be seen, by the experiments here made, that raw beef is applicable to every-day practice in hospitals, cities and places where there is access to markets for the beef. Physicians can prescribe the dose to suit their patients, and it will have to be made fresh every three or four days during warm weather. My experiments were made in July, with the thermometer ranging among the nineties. I judge that in cold weather, this preparation could be made to keep good and sweet for a week or more.

I would suggest a formula for general use, as follows, viz: Fresh raw beef (lean), ℥vi; sweet almonds, deprived of their shells and roasted, ℥i; bitter almonds, ʒvi; sugar, ʒvi; glycerin, ℥ii; water sufficient for emulsion, ℈i. Rub or beat the beef, almonds and sugar to a fine pulp in a wedge-wood or wooden mortar, then add water gradually until a smooth emulsion is formed, and strain through a seive or coarse cloth; return the residuary mass to the mortar, manipulate with the balance of the water until f℥xiv are obtained, strain all through a finer strainer, add the glycerin and bottle; the bottle is to be kept well corked. Dose—f℥i, containing ʒiii of the beef.

The physician, in prescribing, can order the addition of brandy, pepsin, or any other medicine he wishes to administer at the same time. I tried combining ferric pyrophosphate with the mixture; it combines well, but makes a dark, unsightly preparation, on account of the combination of the iron with the blood contained in the beef.

*Chronic Bright's Disease.*—Basham's tincture is always administered. It is the liq. ferri per acetatis, and the formula for its preparation has been given in connection with the reports from Pennsylvania Hospital. The bowels are kept moderately loose by a senna mixture. Diet nourishing. The hot air or vapor-baths are rarely employed when dropsy is present; hydragogues are very much relied upon.

*Treatment of Syphilis according to the Vienna School.*—Zeissl states that, in his experience, he has found that iodine in the proper form will arrest or modify the first manifestations of syphilis, so that only a short course of mercurial inunction will be necessary to effect a cure of the disease. Iodine, when used in this way, will not cause the induration to disappear as rapidly as the mercurials, but it has much more effect upon the skin and mucous lesions. Desirous of avoiding the iodide of potash, he has given iodine in the form of tincture in the following formulæ: Tinct. iodinii, ℨss.; aquæ distill., ℥vi. M. A teaspoonful morning and evening.

Under this method of treatment the lesions of the skin and mucous membranes disappeared in from fourteen to forty-eight days. Iritis and the pustular syphilides were the most obstinate forms of the disease to be met with, and for them mercurial inunction was required. He had never observed any acne follow the treatmeut with iodine. He found iodoform of great use as a local application to indolent ulcers, and also as an internal remedy in the form of pills. The dose should be from two to three grains daily.

Sigmund does not think that the internal use of corrosive sublimate has any advantage over the mercurial unguents; it is necessary, at the same time, to be on the watch for digestive disturbances; the patients must also avoid exposing themselves to taking cold, just as during the treatment by inunction. The sublimate should not be given to old people or young children. He does not believe that there is any advantage in combining the iodide of potash with the mercurials. Psoriasis and certain forms of skin infiltrations are successfully treated by local applications of the sublimate, but there is no special advantage in using it hypodermically; if, however, it is advisable to use some form of mercury in this way—calomel is to be peeferred.—*Wien. Med. Woch.*, 15, 19, 31, 35, 46, 1873.

*Simbat on the Use of Chloride of Zinc in the Treatment of Fistula.*—In a paper published in the *Thèses de Paris*, 1874, No. 73, and based on cases for the most part observed in M. Gaujot's service at the Val-de-Grace, Dr. Simbat shows the good effects which may be obtained by the use of Canquoin's paste in the treatment of fistula, and particularly in the use of anal and even urinary tula. The chloride of zinc is employed with advantage in the treatment of fistula, in consequence of the granulating power it imparts to their walls; by reason of the facility with which it is applied; also in consequence of the absence of the accidents which might accompany wounds from cutting instruments; and, lastly, because it is more likely to prevent recurrence of the evil than other methods of operation.—*London Med. Record.*

As long since as 1859 we employed chloride of zinc in the treatment of fistula in ano. The mode of preparing and using it was suggested by an article published in the *Gazette Medicale de Paris*, by MM. Salmon and Manoury. Our testimony is faverable to its use, especially in cases where the knife cannot be employed, on account of the patient's non-consent, or because he cannot give the time from his ordinary pursuits. For the preparation of this caustic, it is necessary to dissolve some gutta percha in a porcelain cup. When in a state of fusion throw in the determined amount of chloride of zinc, which should be thoroughly incorporated with the gutta percha by means of a spatula. We thus obtain a plastic paste which may be moulded like nitrate of silver, into cylinders, or in sheets like Canquoin's paste. There is nothing more simple, but it is necessary to seize the proper moment to withdraw the mixture from the 6re. This is extracted from the translation we made of the article at the time of its publication. Its mode of application consisted in introducing one of these cylinders into the fistula, and allowing it to remain for a length of time vaiying from two to six hours.—*N. O. Med. und Surg. Journal.*

*Bromide Potassium.*—Dr. Peaslee remarked that he had used bromide of potassium in the treatment of the convulsions of newborn children with good success. But the distinction must be made between this condition and true *trismus*, which occurs later. In the latter condition he does not regard the remedy as at all reliable. There are two general propositions with regard to both effects produced by bromide of potassium.

In the first place, it is a very powerful cerebro-spinal sedative, and exercises but little influence upon the ganglionic nervous system.

In the second place, a similar effect is produced in the part to which it is applied locally.

It is therefore a beneficial agent, used either generally or locally.

He does not regard it as a remedy to be *exclusively* relied upon in the treatment of disease, except in a few instances.

The combination of bromide of potassium with other remedies, in many instances, greatly increases their efficacy. For example: the bromide of potassium will be found to constantly correct the disagreeable effects of opium. In nervous women there is no more valuable remedy than the liq. opii odorata of Dr. Squibb; but there are very many women with whom it does not at all agree. Under such circumstances, if bromide of potassium is combined with the opium, all the evil effects of the opium will be dispelled. When the bromide is used for this purpose, it is necessary to give a sufficient dose to affect the brain. He usually prescribes a single large dose, which is to be taken a few hours before taking the opium.

There are some very anæmic women, upon whom the bromide of potassium will have no effect whatever.

The local application of this remedy in solution is of immense value in some cases of *pruritis vulvæ* and *vagniæ*. First, wash the parts thoroughly with warm water, and then make a topical application of any strength desirable up to a saturated solution. The soothing effect produced is very frequently eminently satisfactory.

In cases of *stomatitis*, *catarrh* of the *posterior nares*, or *pharnyx*, *nausea* and *vomiting* of *pregnancy*, the remedy is very serviceable. In cases of threatened eclampsia, which sometimes occur at the sixth or seventh month of utero-gestation, when the kidneys become inactive, it is one of the greatest safeguards against this complication which we have. Administer it in fifteen gr. doses, t. i. d. When the eclampsia is established it is not to be relied upon.

*Treatment of Acute and Chronic Bronchitis and Asthma.*—Mr. Spurgin, resident medical officer to York Dispensary, employs iodide of potassium in these troublesome complaints (*British Medical Journal*, Sept. 5, 1874). He states: "I have tried iodide of potassium in over a hundred cases with almost invariable success; in fact, with such success, that patients have expressed themselves by saying, 'It has acted like a charm;' others have said that no medicine ever had any real effect upon their complaint before. Iodide of potassium has a marked effect upon breathing, reducing the frequency of the respirations, perhaps (as I think) overcoming spasms. Almost after the first dose, patients have stated they felt the medicine touch their complaint.

"I usually prescribe it with carbonate of ammonia, and, when the cough is very troublesome, add tincture of belladonna and ipecacuanha wine. In the above complaints I rarely give anything else but the above.

"In one case of very severe broncho-pneumonia I tried iodide of potassium, with tincture of hyoscyamus and ammonia, and the respirations were quickly and astonishingly reduced from forty in a minute to less than half that number.

"In conclusion, I should add that I have purposely given a mixture containing ammonia, belladonna, ipecacuanha wine, spirit of sulphuric ether, etc., without iodide of potassium, and have not found much benefit; after which I have added iodide of potassium, and found the patient relieved almost at once."

*Gonorrhœa Mixture.*—Dr. John Morgan recommends the following: ℞. Ac. tannici., ʒi.; spts. etheris nitrici, gtt. xxv.; tr. opii, ʒss.; vin. aromat., ℥iv.; aquæ, ad. q. s., ℥iij. M. Ft. Injec. S. Use every four hours.

*Oxide of Zinc in the Diarrhœa of Infants and Young Children.*—Dr. Brakenridge, of Edinburgh, whose experience is very extensive, and who has employed all the remedies in use for infantine diarrhœa, gives the preference to the oxide of zinc. He says, first: Diarrhœea in these cases arises from a condition of debility and great susceptibility of the nervous centres, which prevent proper secretion from the alimentary tract. 2. It is intimately associated with convulsions and convulsive affections. 3. It is accompanied by congesti[illegible] of the secreting surface of the digestive passages.

To meet these conditions requires a remedy which is at once tonic, antispasmodic and astringent. These properties he believes to be united in the oxide of zinc. It is a tonic for the nervous system, just as iron is for the blood. As an antispasmodic and astringent it has already gained a reputation founded on clinical experience. He has employed it in twelve cases, four of them girls and eight of them boys, and varying in age from four months to one and a half years.. The form was usually that of the powder, but it was also given in a solution of gum-arabic, with a slight addition of glycerin. The general results observed were: 1. That it moderated the diarrhœa quickly. 2. That vomiting stopped. 3. That digestion improved. 4. That intestinal hemorahage was frequentlly arrested. 5. Teething was favored rather than otherwise. 6. That even where no change was made in diet, and the other conditions remained the same, the treatment progressed favorably. 7. When, however, diet and regimen were carefully regulated, success was more rapid and decided.—*Med. Times and Gazette—All. Med. Central-Zeit.*, 47, 1873.

*Emulsion of Cod Liver Oil.*—Mr. W. M. Rice says, in the *American Journal of Pharmacy*, November, 1873, on this preparation:

After a series of experiments, at the request of, and assisted by, a medical friend, the writer of this has perfected the following formula, which he offers to his professional brethren, hoping it may prove useful: ℞.—Oleum morrhuæ, fl.℥viij.; tragacanth, ʒj.; sacchar. alb., ʒiv.; ol. gaultheriæ, gtts. lx.; ol. sassafras, gtts. l.; ol. amygd. amar., gtts. x.; aquæ, fl.℥viij.

The tragacanth and sugar are to be dissolved in the water, and the mucilage strained. In this is to be incorporated, first, the essential oils, and then the cod liver oil. This makes an elegant-looking emulsion, not too thick, containing fifty per cent. of the oil, and of a rather pleasant taste and smell.

Many manufacturers combine the lacto-phosphate of lime, etc., with the cod liver oil mixture; but as physicians often consider this decidedly objectionable in a medicine intended, as this is, in most cases, for continued use for a considerably protracted length of time, the author has been induced to omit it. It can be added, however, by a slight modification of the above formula.

*Chloral in Threatened Miscarriage*—Dr. Martineau lately stated at a meeting of the Society of Therapeutics of Paris that a woman, seven months and a half pregnant, was admitted last year under his care at the Hotel Dieu. She was suffering from ague, and was treated by sulphate of quinine. Uterine pains came on, either from the effect of the quinine or the intermittent fever, and to prevent miscarriage an enama with laudanum was administered. This was of no avail; but a few enemata of hydrate of chloral were effectual—the contractions ceased, and eventually normal parturition took place on the 15th of March last. The chloral was also successful in the case of a patient of M. Martineau's, four months and a half in the family way. She had an attack of pleuro-pneumonia of the right side, and was twice cupped. Four days afterwards pains occurred, and a red flux was taking place from the vagina. M. Martineau ordered an enema of fifteen grains of hydrate of chloral to four ounces of water, and three such enemata were given at twelve hours' interval. These had the desired effect; the pneumonia was cured in nine days. No miscarriage had taken place, and the patient again felt the movements of the child.—*London Lancet.*

*Burns.*—The following prescription has been employed, and its use has been attended with very gratifying results:

℞. Tannic acid, ℈i; chloroform, gtt. xx; simple cerate, ℥i. M. Spread upon lint and cover the parts affected.

Glycerin and bismuth have also been somewhat employed of late, but with the result of producing terrible smarting when first applied, usually continuing for an hour or more.

Bismuth and glycerin are united in such proportions as will give a creamy paste which can be conveniently spread upon sheet lint. It is possible that the glycerin was impure, or the bismuth was not an impalpable powder, or both circumstances may have united in producing an inordinate amount of local irritation.

When the burn is deep, such dressings are employed as may be of service in assisting the separation of the slough; when the slough has separated, the excavation is treated as an open ulcer.

*Administration of Perchloride of Iron.*—Dr. H. L. Snow states (*British Medical Journal*, June 28, 1873) that the astringent metallic taste long remaining in the mouth after the administration of tincture of the chloride of iron, the flavor of which is not very imperfectly disguised by the syrup or spiritus chloroformi with which it is usually ordered, may be altogether obviated by the substitution of a small quantity of glycerin (about half-an-ounce to an eight-ounce mixture).

*Phenic Acid in the Treatment of Cutaneous Affections.*—Professor Doutrelepont recommends phenic acid in prurigo, pruritis, and especially in psoriasis. In eczema he found it useful only to relieve the intolerable itching which accompanies this affection, and to diminish the disposition of the patient to scratch himself.

In one case of psoriasis of fifteen years' duration in which the entire surface of the body was affected, the prolonged administration of phenic acid internally, and in strong doses, effected a complete cure, and the author hopes in continuing the medicine in small doses to be able to prevent a return of the affection. The quantities of phenic acid which Prof. Doutrelepont prescribes are quite considerable. He gives 40 grains divided in several doses through the day. He has never, as a consequence, seen albumen in the urine, nor recognized any kind of inconvenience from it. Still, he ordinarily commences with feeble doses, giving, for example, seven grains a day in the form of pills.—*Bulletin de Thérapeutique.—Annales de Dermatologie et de Syphiligraphie.* No. 5, 1874.

*Tincture Eucalyptus Globules in Intermittent Fever.*—The following results are summed up by Dr. Hirsch, (*Berl. Klin. Wochenschrift*, No. 30,) as obtained from his experiments with the tincture in nine cases of obstinate intermittent fever:

1. In all the cases, after the use of the remedy for one or more days, the spleen diminished in size.

2. In six cases, three, at most four, teaspoonfuls of the medicine were sufficient to prevent a return of the paroxysms. In one case only was the double quantity required.

3. Seven of the nine cases were cured completely; in the remaining two the remedy proved unsuccessful.

From these results Dr. H. draws the conclusion that tinct. eucalypt. glob. is a remedy but little, if any, inferior to quinine in the treatment of intermittent fever, and that it will probably prove to be as valuable an antiphlogistic in the treatment of other fevers as quinine, digitalis and veratrum.

*Facial Erysipelas*—Erysipelas had been in the hospital to quite an extent. For local application : saturated solution of *sulphite* of soda. Constitutional treatment: ℞. of iron gtts. xx. every two hours during the day; and gtts. xxx. every three hours during the night; quinine, grs. xvi. during twenty-four hours, in doses of two grains. A most terrible-looking case of what was denominated "erratic erysipelas" was seen in a patient far advanced in phthisis. Extensive phlegmonous inflammation of the scalp had been developed. The patient was doing remarkably well under the influence of the soda dressing, iron, quinine and milk punch, of which latter tonic as high as ℥ xl. had been administered during twenty-four hours.

*Cosmetic Surgery.*—A few weeks ago we made a note on the value of benzine in removing pimples, comedones, etc. Ether may also be used for the same purpose. The pimples should be washed several times, till the sebum is dissolved, and subsequently a solution employed as a toilet lotition.

*The Druggist* says, "For the benefit of young persons afflicted with freckles, it may stated that powdered nitre, moistened with water, applied to the face night and morning, will soon remove all traces of them."

Dr. James Nicholls writes to the *Lancet*, strongly recommending the use of carbolic acid in removing nævi, moles, and other congenital discolorations of skin. He has tried it in several aggravated cases with most successful results.

For boils, Dr. Hall, in the Cincinnati *Lancet and Observer*, recommends the following: ℞—Tr. arnica flowers, ℥j.; tannic acid, ℥ss.; gum acacia pulv., ℥ss.—M. It should be used as soon as prepared. The inflamed surface, and a little beyond all round, should be painted with the medicine every fifteen minutes, or as fast as it dries, till a good thick coating covers the part.

*Local Trertment of Pulmonary Cavities.*—Dr. Mosler, of Griefswald, has, in a number of cases, and with relative success, penetrated directly from the surface through the thoracic parietes into caverns, pulmonary abscesses, and bronchial dilatations, treating them like ordinary abscesses.

One or more punctures are made with a canula of sufficent size, and a small quantity of permanganate solution injectd. Dr. Mosler tdinks that the pulmonary parenchyma is more tolerant than is generally supposed, and that it may be acted upon through the thoracic parietes in the same manner as any other organ to which the surgeon has access. Not only pulmonary cavities, but pulmonary inflammations may be treatéd by medicated injections, just as other neoplastic parenchymatous tumors are treated. — *Berliner klinische Wochenschrift—New York Medical Journal.*

*Test for Impurities in Water.*—An English technical periodical says: "Good water should be free from color, unpleasant odor and taste, and should quickly afford a lather with a small portion of soap. If half a pint of the water be placed in a perfectly clean colorless glass-stoppered bottle, a few grains of the best lump white sugar added, and the bottle freely exposed to the daylight in the window of a warm room, the liquid should not become turbid, even after exposure for a week or ten days. If the water become turbid, it is open to the grave suspicion of sewerage-contamination ; but if it remain clear, it is almost certainly safe. We owe to Heisch this simple, valuable, but hitherto strangely neglected, test."

*Treatment of Furuncles.*—Prof. Hardy, when boils appear in various parts of the body, almost always succeeds in dispersing them by general treatment. He never employs, as is too often done, frequent purgatives, and very rarely practices incision. If this last be employed too soon, it does not cure the furuncle, always leaving an indurated base which ends in a mortification of the cellular tissue. Made too late, the incision is useless. Among all the various internal remedies that have been recommended, he prefers tar, ordering about a quart of tar-water to be drunk daily. In some cases alkalines or arsenical preparations may be used, or the sulphurous mineral waters of Aix, or Bagnéres-de-Luchon. When there is a complication with dyspepsia, alkaline waters, such as those of Vichy, are indicated.—*Union Méd.*, September 1, 1874.

*Treatment of Gonorrhœa.*—The following is an extract from a lengthy article by Dr. Haberkorn, in the *Berl. Klin. Wochenschrift*, No. 34, on the above subject:

Injections of permanganate of potassa, carbolic acid, sulphate of zinc, and other remedies, have all proved more or less insufficient in the treatment of gonorrhœa. After repeated experiments the author has found the sulphate of quinine to be a far superior remedy, being prompt in its action and nearly painless. He directs about a teaspoonful of the following mixture to be injected three times a day, retaining it for some time in the uretha: ℞. Quiniæ sulphat., gr. xv.; acid. sulphur., dil. ℈j.; glycerinæ, f℥vj.; aquæ, f℥ij. After three days a great improvement took place in all his cases. The expense of the medicine is covered by the rapidity of the cure. These results therefore justify a more extensive trial of this remedy.

*Thudicum's Douche.*—Dr. Frederick P. Henry (*Phila. Medical Times*, May 17, 1873) mentions the case of a girl, two years old, who had introduced a large-sized shirt-button into her nose, and was quickly relieved by the aid of Thudicum's douche. The child's cries, which were comprised in one prolonged expiratory effort, seemed to have aided the operation by raising the velum, and thereby preventing escape of water into the mouth, which any one who has used the instrument on his own person knows is so liable to occur.

*A New Diaphoretic.*—At a meeting of the Société de Biologie, on the 12th of April last, Rabuteau and Couthino made a communication on the subject or jaborandi, a Brazilian plant of the order Rubiaceæ, which has remarkable diaphoretic properties. Rabuteau took forty-eight grains, and fifteen minutes afterwards began to sweat freely, and had copious discharges of saliva. A bitter principle seemed to be the essential chemicai constituent of the leaves.—*Berl. klin. Woch.*, 30, 1874.

VAGINITIS.—Prof. T. D. Fitch, *Chicago Med. Ex.*) says: In the acute form use warm sitz baths, mucilaginous vaginal injections, and give saline laxatives; if pain is severe give anodynes. A very excellent formula for a vaginal injection, and the one that I am in the habit of prescribing, is pot. chloras, ʒiv.; pot. permang. gr. x.; aquæ, ℥xvj. M. A teacupful morning and evening, with a little warm water added.

Another very excellent plan is the use of cotton tampons of glycerite of tannic acid.

Introducing one of these tampons every three or four days, by attaching a string to them, the patient can very readily remove them herself. In using them you should be careful to caution the patient about the liability of staining her clothing from the tannin, in order that she may wear an appropriate bandage.

*Amyl Hydride.*—The amyl hydride was exhibited to the Society, and was considered to be innocuous when applied to the skin or mucuous membranes. If swallowed it is quickly converted into vapor. It is a remarkable solvent.

A solution containing one scruple of iodine to the ounce of amyl hydride, is very simple, painless, and effective, when applied to bad smelling sores, ulcers, wounds, and to cancer: a very quick disinfectant. Common oils, spermaceti, and other similar oily substances, make ready solutions in amyl hydride.

Burns may be treated with success by using such a solution. *Med. Ex.*

*Dressing with Magnesia.*—Dr. Ohleyer advocates, in *Allgemeine Med. Cent. Zeitung*, No. 47, 1873, the use of magnesia, which he has found very successful in the dressing of certain ulcers when fermentative processes retarded healing. Magnesia neutralizes the acids present, prevents the access of oxygen to the surface, and protects the granulations. The author especially applies it to (1) atonic ulcers: (2) cases in which the skin is without epidermis and in which there is danger of suppuration; (3) inflamed and painful sores; (4) wounds which require to be stimulated or to be withdrawn from the influence of air, or in which suppuration should be diminished or modified. Dr. Ohleyer has also used magnesia with good results in erysipelas of the face, as an isolating substance. *Lancet*, July 12, 1873.

*Retention of Urine Relieved by Ice in the Rectum.*—M .Cazonave's plan is to plug the rectum with small pieces of ice. Mr. Teevan relates a case in which he adopted this procedure In exactly twenty minutes after the last fragment had been inserted into the bowel the patient began to pass water guttatim, and in the course of half an hour contrived to empty his bladder.—*Mr. W. F. Teevan.*

*Puerperal Mania; Treatment by Choral Bromide of Potassium.*—The patient, aged thirty years, had been suffering severe anxiety, previous to and during labor, from some domestic trouble. The position was transverse, and delivery accomplished by version. Following the labor were severe after-pains, for which morphia was administered. That night the pulse ran up to 130 per minute, the temperature to 102½°, and with this fever marked delirium set in. The delirium continued for two nights and one day, when the treatment, which had been morphia with veratrum viride, was changed to bromide of potassium with hydrate of choral. Two hours after the latter remedies had been administered, the patient slept, and on awaking was perfectly rational. This improvement continued.—*N. Y. Med. Journal.*

*Delirium Tremens.*—The standard prescription for this condition at the Roosevolt Hospital, New York, is: ℞.—Chloral hydrat., grs. xxx.; potass. brom., grs. xl., to be given at bed time, and continued through the day in smaller doses, if necessary.

*Acute Articular Rheumatism.*—At the Charity Hospital, New York, the following is in use as a local application: ℞.—Tinct. opii, ℥i.; spiritus chloroformi, ℥iss.; liniment sapodis, ad oi.—M. This liniment is applied freely to the joints and immediately covered with cotton and oiled silk. The relief from pain afforded by this application has been gratifying.—*New York Medical Record.*

*Lumbago.*—If internal remedies, such as iodide of potassium and opium, have had no appreciable effect, apply a continuous galvanic current, at the same time making the patient exercise the affected muscles rythmically, by bending, extending and rotating the spine. From 10 to 18 cells of Mayer and Meltzer's zinc carbon battery should be employed. The positive pole is to be placed at the upper part of the spinal column, in the middle line, and the whole back, including the intercostal muscles, is to be sponged with the negative pole.—*Dr. G. V. Poorer, Lancet, December 27th.*

*Hemorrhoids.—The Elastic Ligature.*—This mode of operating is especially adapted for hemorrhoidal tumors, nævi, and warts, and I should have used it more frequently than I have done had not experience proved the superiority of the curved clamp and the actual cautery where there is no objection to their use.—*Mr. Hy. Lee, Medical Times, December 6th.*

Dr. Richardson's new "tooth-edged cutting scissors" are an admirable instrument for snipping a groove at the lower edge of internal hemorrhoids prior to ligaturing them, as no bleeding occurs, andthe view of the part is consequently not obscured.—*Mr. C. F. Maunder.*

